# Advances in the Diagnosis and Treatment of Acanthamoeba Keratitis

**DOI:** 10.1155/2012/484892

**Published:** 2012-12-06

**Authors:** Benjamin Clarke, Arti Sinha, Dipak N. Parmar, Evripidis Sykakis

**Affiliations:** Department of Ophthalmology, Whipps Cross University Hospital, Whipps Cross Road, London E11 1NR, UK

## Abstract

This paper aims to review the recent literature describing Acanthamoeba keratitis and outline current thoughts on pathogenesis, diagnosis, and treatment as well as currently emerging diagnostic and treatment modalities.

## 1. Introduction

Acanthamoeba keratitis (AK) has been identified as an entity since 1973 when the first case of an American patient was reported to an ocular microbiology group in Dallas. The first published reports emerged in the UK in 1974 [1]; the two cases required corneal grafting and enucleation, respectively, with retrieved tissues from both cases found to have acanthamoebic cysts when examined with light microscopy. Since the pathogenicity of Acanthamoeba towards the eye has been recognised, case numbers have risen [2]. This actual rise has been shown to be related to increased rates of contact lens wear [3], in particular soft lenses and the use of one-step cleaning solutions, which may allow survival of increased numbers of amoebae [4].

Extensive reviews of the subject have been published by Illingworth and Cook in 1998 [5], Hammersmith in 2006 [6], and Dart et al. in 2009 [7] since there have been exciting new developments in our understanding of the disease, with new techniques for diagnosis and treatment emerging as realities. The aim of this paper is to bring the reader up to date with current and emerging practices.

## 2. Epidemiology and Pathogenesis

Acanthamoeba is one of three amoebic parasites that are thought to be significant to human disease, amongst Entamoeba (responsible for amoebic dysentery) and Naegleria (responsible for amoebic meningoencephalitis). Acanthamoeba is known for causing keratitis or granulomatous amoebic encephalitis (GAE). Found naturally in soil and fresh water, it can exist in the pathogenic trophozoite form, or in times of physiological stress, it will encyst and become metabolically dormant. The cystic form is very resistant to chemical injury or desiccation, which makes it harder to treat than other microbes. Acanthamoeba has been found to colonise the nasal mucosa in up to 24% of environmentally exposed populations [8], although its pathogenic activity is much more rare. Acanthamoeba keratitis has a large variation in reported rates between countries, which is thought to be largely due to differences in diagnostic criteria, rather than differences in populations. Schaumberg et al. found a US incidence of 2 cases per million contact lens wearers in the late 1980s [9] as compared to over 21 cases per million contact lens wearers in the UK in 1998 [10]. Whether this growth represents an increased accuracy in diagnosis, a shift in the habits of contact lens wearers, or indeed a combination of the two, we have to accept that Acanthamoeba keratitis is becoming an increasingly significant problem.

In 1977, Pussard and Pons [11] identified three distinct groups of Acanthamoeba (1–3), based on microscopic appearance of the encysted organism. It has been shown that groups 2 and particularly 3 are more virulent in human keratitis. Evidence has also been emerging that epithelial infection with acanthamoeba is augmented by bacterial or viral coinfection [12], with contact lenses providing a platform for both organisms to simultaneously set to work on the cornea.

Contact lens solutions have been coming under an increasing scrutiny for allowing acanthamoeba survival. Hiti et al. [13] found that all-in-one solutions were inferior to two-step cleaning regimes at eradicating encysted organisms of two separate pathogenic strains, raising concerns for the current recommended practice for contact lens hygiene.

Contact lens wear is not always the main risk factor for infection, however. In a recent epidemiological study from India [14], only 0.9% of reported cases of AK were thought to be associated with contact lens wear. The major risk factors were associations with eye trauma and poor water supply.

Whilst GAE has only been reported in immunocompromised individuals, keratitis may affect those who are healthy and immunocompetent. All normal individuals will mount a humoural immune response to the infection, which is effective in vascularised regions of the body. However, in the immunoprivileged cornea, the normal oxidative destruction of the organism by neutrophils is weakened as it relies on antibodies marking the organism prior to destruction.

The pathogenesis of the keratitic process has been classified into three stages: (1) epithelial adhesion and desquamation; (2) stromal invasion; (3) neuritis. 

### 2.1. Epithelial Adhesion and Desquamation

Ancanthamoeba has been observed to adhere to healthy epithelium, without a traumatic entry point [15]. This is facilitated by glycoproteins and glycolipids present on human corneal epithelial cells, which are believed to interact with a 136kDa mannose binding protein that is expressed on Acanthamoeba cells membranes [16]. The amoebae are able to burrow under epithelial cells, where they cause rapid desquamation through three mechanisms: direct epithelial cell cytolysis; phagocytosis; induction of apoptosis. 

### 2.2. Stromal Invasion

A combination of lytic enzymes allows trophozoites to invade the extracellular matrix of stromal cells, gain access to stromal tissue, and induce the ring infiltrate seen in clinical infection. Serine proteases, a metalloproteinase, a cysteine protease, an elastase, a collagenolytic enzyme, and a plasminogen activator have all been associated by in vitro studies [17].

### 2.3. Neuritis

Trophozoites have been shown to follow a chemotactic response to corneal neurones and may cause a cytolytic and apoptotic response, causing the clinical sign of radial neuritis. In the majority of cases, this is the final stage of inflammation in the clinical setting. Trophozoites have not been found to disrupt corneal endothelial cells and enter the anterior chamber in vivo despite the cytolytic and aopototic effect in vitro. Subsequently, cases of Acanthamoeba endophthalmitis are rare [18].

In Entamoeba, which has prolific capability to invade epithelial cells, a plasma membrane associated collagenase is associated with virulence of the organism. No such marker has been found for Acanthamoeba, although phospholipase activity may be a candidate [19].

## 3. Diagnosis

Clinical symptoms are often corneal pain and photophobia, which may be disproportionate to the appearance of the eye. Initial findings are often punctate epitheliopathy or scattered subepithelial infiltrates which respond well to steroid therapy. The recognised pathognomonic sign of AK is a radial pattern of perineural infiltrates, often with an associated limbitis. Ring infiltrates are common, with a variable onset from early in the infection until very late. Advanced stages show a central epithelial loss with stromal thinning and occasionally progression to corneal melt. Uveitis and hypopyon may occur in the later stages of the infection. The common disciform epithelial and stromal infiltrate appearance causes Acanthamoeba keratitis to be very commonly initially diagnosed as herpes simplex virus (HSV) keratitis or even fungal keratitis, until treatments for these conditions fail to effectively treat the patient.

Clinical suspicion is the first and most vital step in managing Acanthamoeba. A detailed clinical history will usually reveal risk factors—either contact lens wear in western countries or trauma and water exposure in the developing world. Despite this, the early clinical appearance will usually mimic HSV keratitis, and thus most patients undergo treatment for this in the first few weeks or months. Failure to respond swiftly to antiviral or antibacterial therapy should always raise the suspicion of acanthamoeba. Amoebic cultures should be ordered for any corneal scrape where there is clinical suspicion and every time when a repeat scrape needs to be taken due to lack of growth. Acanthamoeba feeds readily on an inactivated E coli set on an agar plate, and cultures need to be checked under a light microscope daily for trails that indicate migration of Acanthamoeba. Cultures are used in conjunction with a smear slide for microscopy and often a small corneal lamellar disc biopsy, taken under local anaesthetic. These may be examined by staining with with calcofluor-white or immunoperoxidase [20] to aid in the detection of the trophozoites or cysts. Recent advances in immunohistological staining such as Acanthamoeba specific monoclonal antibodies reported by Turner et al. [21] have added to the precision in which Acanthamoeba may be detected by traditional laboratory methods.

Polymerase chain reaction (PCR) amplification has been used since 1996 in detecting Acanthamoeba, and a recent study on its accuracy by Boggild et al. [22]showed that it compared favourably with smear microscopy and biopsy histology in terms of sensitivity, although specificity was still slightly poorer. More recently, reports have been emerging such as those by Kandori et al. [23], Itahashi et al. [24], and Ikeda et al. [25], which demonstrate the use of real-time quantitative PCR for Acanthamoeba detection, eliminating the need for gel-based electrophoresis and time-consuming processing. Khairnar et al. [26] compared real-time PCR to gel-based techniques, along with traditional smear and biopsy microscopy, found similarly higher sensitivity of 89.3% in the real-time technique, and suggested that both PCR methods are favourable to the traditional techniques. Further refinements in the PCR technique have also been developed to yield higher sensitivities. Le Calvez et al. [27] recently described a multiplexed quantitative PCR method which is able to distinguish individual Acanthamoeba species in free-living mixed flora samples. Laummaunwai et al. [28] developed an algorithm for DNA extraction which is able to produce viable DNA from a single Acanthamoeba cyst; previously cystic DNA extraction has been the Achilles heel of the technique. These advances mean that real-time PCR is becoming more and more relevant in diagnostics, with particular benefits being that it is a much faster technique than growing Acanthamoeba cultures and does not require adjuvant biopsy, so it can be implemented earlier, on cases of lower clinical suspicion.

H1 nuclear magnetic resonance (NMR) spectroscopy has recently been used to identify Acanthamoeba in vitro by Hauber et al. [29]. By profiling the biochemical signature of different strains of the organism, it is anticipated that this method could yield a high level of sensitivity and specificity as a diagnostic test. Its application would be similar to PCR testing, so further study is needed to see how it compares to PCR in terms of diagnostic accuracy and time efficiency.

**Figure 1:**
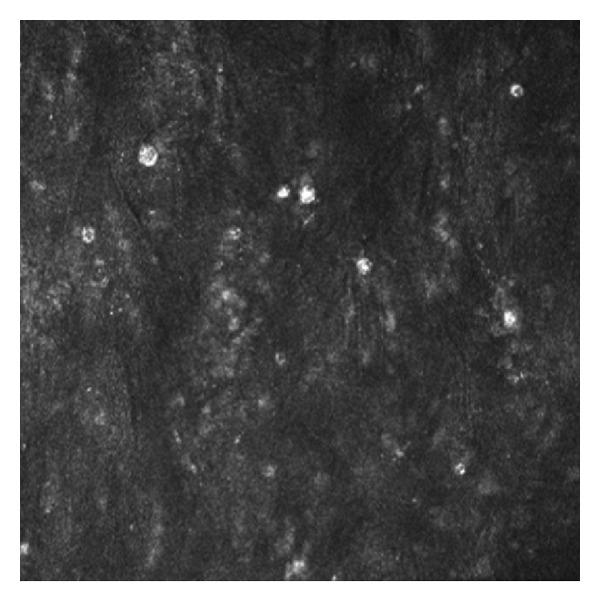
In vivo corneal confocal microscopy: amoeba cysts seen as round hypereflective lesions.

Confocal microscopy has recently become a powerful tool for rapid diagnosis of the infection in vivo, and without the need to wait for culture and microbiological analysis. The obvious advantage of in vivo microscopy is that biopsy is not needed and the diagnosis can be instantaneous in the hands of an experienced operator on observation of the round hypereflective lesions of amoeba cysts (see Figure 1). In 2006 Parmar et al. [30] studied 63 AK suspected cases and demonstrated that in vivo corneal tandem scanning confocal microscopy (TSCM), apart from being rapid and noninvasive, was much more sensitive than either culture or biopsy analysis in Acanthamoeba. This was thought to be due to the increased resolution that TSCM is able to provide, by filtering out reflected light, down to resolutions of 1-2 *μ*m laterally and 5–10 *μ*m axially. Diagnostic accuracy has been studied more recently, and in 2010, Hau et al. [31] looked at a laser confocal system as used by one person, but with images graded by different observers. This showed a wider range of accuracy, but sensitivity was recorded as high as 55.8% and specificity up to 84.2%. In 2011 Vaddavalli et al. [32] produced higher values of 88.3% sensitivity and 91.1% specificity, similar values to Tu et al. [33] in 2008. Interobserver agreement was comparable to Hau et al., but was however calculated in a less pragmatic manner. We can therefore assume that in a real clinical environment with multiple observers and graders, the accuracy may lie closer to Hau et al.'s values, which would nonetheless give a very useful adjunct to clinical examination and should now be considered a first-line method in Acanthamoeba diagnosis where facilities exist.

## 4. Treatment

Treatment regimes reported in the literature have varied widely depending on the extent of the disease being reported, the general health of the cornea, and the personal experience of the physician. In the original case report by Naginton et al. [1], numerous topical antimicrobial preparations were tried in conjunction with steroids, but both eyes eventually required grafting. Recent years have brought us knowledge of more specific antimicrobials, although the failsafe remains the surgical grafting of the cornea.

Topical therapies will usually involve a Biguanide (polyhexamethylene biguanide (PHMB) 0.02% or chlorhexidine 0.02%) in combination with a diamidine (propamidine is ethanoate 0.1% or hexamidine 0.1%) initially at a high frequency. Hourly drops may be tapered down after 48 hours to alleviate the epithelial toxicity caused by both these compounds. Topical therapy for AK needs to be continued much longer than antibacterial therapy regimes due to the encystment of the amoebae, which is much harder for the drugs to penetrate. Typical regimes will taper over around 6 months.

Recent case reports have pointed towards triazoles as an adjunct to biguanide and diamidine therapy in refractory cases. Conventionally used as antifungals, they have been used empirically in AK. Bang et al. [34] reported in 2009 a marked improvement in three eyes treated with topical voriconazole 1% drops in addition to standard therapy. Tu et al. [35] found similarly promising results in 2010 using oral voriconazole 200 mg twice daily, but noted that extended duration treatment was required in order to prevent relapse.

Steroids have always provoked controversy, as with other forms of keratitis. Current thinking is that a topical steroid may be added once a sterilisation period of antimicrobial therapy has been completed, in some cases, several weeks. Park et al. [36] studied the use of steroids in AK treatment, and how this is related to visual outcomes. They found no link between starting steroids (even before antiamoebal treatment) and worsened visual outcomes. The only caveat was that late initiation of steroids might prolong the life cycle of resistant cysts, thus prolonging the duration of treatment required. Thus, introduction of steroid therapy may be prudent in the early stages of disease if clinically indicated.

Surgery may be required in cases where the cornea is permanently scarred or in cases refractory to maximum medical treatment. Awwad et al. [37] in 2005 described penetrating keratoplasties (PK) in thirteen quiet eyes at least three months after stopping amoebacidal treatments for AK. Final visual acuities ranged from 20/15 to 20/40, and no episodes of rejection or disease reactivation were recorded. Whilst a good outcome can be expected in eyes that have responded well to treatment, it is of course a larger challenge to maintain a graft in an eye with an ongoing infection. Nguyen et al. [38] reported 9 such cases with final acuities between 20/15 and 20/50 with no recurrences after 17 months of followup. In 2007, Parthasarathy and Tan [39] reported a case of deep lamellar keratoplasty (DLK) for treatment of refractive AK in 2007, with the patient eventually retaining 20/20 vision. This has the obvious advantage of maintaining an intact globe intraoperatively, which serves to reduce intraocular entry of organisms and maintains an intact endothelium, which may improve graft survival. This has since been incorporated into clinical practice. In an outbreak of AK in Singapore reported in 2009 by Por et al. [40], 11 out of 43 eyes required therapeutic DLK, and one required therapeutic PK. Recurrence of disease was seen in one DLK, which required further PK surgery. Final visual acuities were again mixed, with only 25 of the eyes obtaining 20/40 or better. Szentmáry et al., in a recent review [41], reported improved outcomes of keratoplasty in those procedures performed after three months of keratitis inactivity, suggesting that surgery should be performed later in the clinical course if possible.

Recently, the widespread use of photorefractive surgery has inspired its use in the setting of AK. Kandori et al. [42] reported four cases in 2010, where early stage AK was treated with standard topical therapy, but developed large corneal abscesses in the upper third thickness of stroma. These were removed using laser phototherapeutic keratectomy (PTK); all eyes experienced no disease recurrence and final acuities ranged from 20/16 to 20/25. This would seem to be a very promising modality, although its application to more deeper or more widespread infiltration may be limited.

Cross-linking is another relatively new treatment option that has been applied to AK. Whilst in vitro studies by Kashiwabuchi et al. [43] and del Buey et al. [44] have shown no amoebacidal effect of riboflavin combined with UVA exposure, clinical case reports have shown a much more promising picture. Garduño-Vieyra et al. [45] administered collagen cross-linking to a patient in Mexico in place of topical medical therapies, which were not commercially available. A significant improvement was observed after 24 hours, with symptoms resolving within three months, and a 20/20 vision was obtained after five months. Khan et al. [46] have since reported three similar cases which responded equally well to cross-linking, with all ulcers closing within seven weeks. In subsequent PK surgery for scarring, no organisms were detected in excised tissue. It is possible that the collagen stabilising effect prevents further tissue damage [47] and isolates and prevents reproduction of the amoebae. Although individual case reports results seem promising, there are no formal clinical trials thus far to recommend incorporation into standard practice.

## 5. Conclusion

Acanthamoeba keratitis is a potentially devastating disease that, although rare, constantly presents difficulties in diagnosis and treatment. Since the first cases in 1973, we have expanded our knowledge of the clinical manifestations of the disease and have come to recognise them. Recent advances of PCR and confocal microscopy have started to improve our diagnostic ability greatly, and as they become more recognised and available, it is hoped that they will serve us more in clinical practice. Treatment still relies on topical biguanides and diamidines as the mainstay of treatment for straightforward cases, but we have also learnt that steroids may be used safely in cases with a significant inflammation. Surgery has been necessary for resistant disease, and we have seen that DLK may provide good outcomes whilst maintaining the host endothelium. Laser photokeratectomy may become more important as we continue to explore its indications. Cross-linking needs further study, as initial case reports show promise, as a useful adjunct to surgery, or possibly even a treatment modality in its own right.
